# Ultrasound Muscle Evaluation for Predicting the Prognosis of Patients with Head and Neck Cancer: A Large-Scale and Multicenter Prospective Study

**DOI:** 10.3390/nu16030387

**Published:** 2024-01-29

**Authors:** Rocío Fernández-Jiménez, Silvia García-Rey, María Carmen Roque-Cuéllar, María Luisa Fernández-Soto, María García-Olivares, María Novo-Rodríguez, María González-Pacheco, Inmaculada Prior-Sánchez, Alba Carmona-Llanos, Concepción Muñoz-Jiménez, Felisa Pilar Zarco-Rodríguez, Luis Miguel-Luengo, Hatim Boughanem, Pedro Pablo García-Luna, José Manuel García-Almeida

**Affiliations:** 1 Department of Endocrinology and Nutrition, Virgen de la Victoria University Hospital, Málaga Biomedical Research Institute and BIONAND Platform (IBIMA), 29010 Malaga, Spain; rociofernandeznutricion@uma.es (R.F.-J.); jgarciaalmeida@uma.es (J.M.G.-A.); 2 Department of Endocrinology and Nutrition, QuironSalud Malaga Hospital, 29004 Malaga, Spain; 3 Department of Medicine and Dermatology, Faculty of Medicine, University of Malaga, 29010 Malaga, Spain; 4 Department of Endocrinology and Nutrition, Virgen del Rocío University Hospital, Instituto de Biomedicina de Sevilla (IBIS), 41013 Seville, Spain; silviam.garcia@juntadeandalucia.es (S.G.-R.); mariac.roque@juntadeandalucia.es (M.C.R.-C.); pgarcia1@us.es (P.P.G.-L.); 5 Endocrinology and Nutrition Clinical Management Unit, University Hospital San Cecilio, 18012 Granada, Spain; mlfernan@ugr.es; 6 Biosanitary Institute of Granada, Medicine Department, Faculty of Medicine of Granada, University of Granada, 18010 Granada, Spain; 7 Department of Endocrinology and Nutrition, Instituto de Investigación Biomédica de Málaga (IBIMA), Regional University Hospital, 29007 Malaga, Spain; 8 Department of Endocrinology and Nutrition, FIBAO (Fundación para la Investigación Biosanitaria de Andalucia Oriental), Virgen de las Nieves University Hospital, 18014 Granada, Spain; marianovor@correo.ugr.es; 9 Department of Endocrinology and Nutrition, Instituto de Investigación e Innovación Biomédica de Cádiz (INIBICA), Puerta del Mar University Hospital, 11009 Cadiz, Spain; maria.gonzalez@inibica.es; 10 Department of Endocrinology and Nutrition, Jaen University Hospital, 23071 Jaen, Spain; mariai.prior.sspa@juntadeandalucia.es; 11 Department of Endocrinology and Nutrition, Instituto de Investigación e Innovación Biomédica de Cádiz (INIBICA), Jerez de la Frontera University Hospital, 11407 Cadiz, Spain; alba.carmona@inibica.es; 12 Department of Endocrinology and Nutrition, Maimonides Institute for Biomedical Research in Córdoba (IMIBIC), Reina Sofía University Hospital, 14011 Cordoba, Spain; munoz.jimenez@sspa.juntadeandalucia.es; 13 Department of Endocrinology and Nutrition, Valme University Hospital, 41014 Seville, Spain; felisa.zarco.sspa@juntadeandalucia.es; 14 Department of Endocrinology and Nutrition, Badajoz University Hospital, 06080 Badajoz, Spain; luismluengo@unex.es; 15 Departamento de Ciencias Biomédicas, Facultad de Medicina, Universidad de Extremadura, 06006 Badajoz, Spain; 16 Spanish Biomedical Research Center in Physiopathology of Obesity and Nutrition (CIBERObn), Instituto de Salud Carlos III, 28029 Madrid, Spain; 17 Unidad de Gestión Clinica Medicina Interna, Lipids and Atherosclerosis Unit, Maimonides Institute for Biomedical Research in Córdoba (IMIBIC), Reina Sofia University Hospital, University of Córdoba, 14004 Cordoba, Spain

**Keywords:** RF-CSA, muscle mass, malnutrition, survival, oropharyngeal cancer, sarcopenia, random forest, decision tree

## Abstract

Head and neck cancer (HNC) is a prevalent and aggressive form of cancer with high mortality rates and significant implications for nutritional status. Accurate assessment of malnutrition in patients with HNC is crucial for optimizing treatment outcomes and improving survival rates. This study aimed to evaluate the use of ultrasound techniques for predicting nutritional status, malnutrition, and cancer outcomes in patients with HNC. A total of 494 patients with HNC were included in this cross-sectional observational study. Various tools and body composition measurements, including muscle mass and adipose tissue ultrasound evaluations, were implemented. Using regression models, we mainly found that high levels of RF-CSA (rectus femoris cross-sectional area) were associated with a decreased risk of malnutrition (as defined with GLIM criteria (OR = 0.81, 95% CI: 0.68–0.98); as defined with PG-SGA (OR = 0.78, 95% CI: 0.62–0.98)) and sarcopenia (OR = 0.64, 95% CI: 0.49–0.82) after being adjusted for age, sex, and BMI. To predict the importance of muscle mass ultrasound variables on the risk of mortality, a nomogram, a random forest, and decision tree models were conducted. RF-CSA was the most important variable under the random forest model. The obtained C-index for the nomogram was 0.704, and the Brier score was 16.8. With an RF-CSA < 2.7 (AUC of 0.653 (0.59–0.77)) as a split, the decision tree model classified up to 68% of patients as possessing a high probability of survival. According to the cut-off value of 2.7 cm^2^, patients with a low RF-CSA value lower than 2.7 cm^2^ had worse survival rates (*p* < 0.001). The findings of this study highlight the importance of implementing ultrasound tools, for accurate diagnoses and monitoring of malnutrition in patients with HNC. Adipose tissue ultrasound measurements were only weakly associated with malnutrition and not with sarcopenia, indicating that muscle mass is a more important indicator of overall health and nutritional status. These results have the potential to improve survival rates and quality of life by enabling early intervention and personalized nutritional management.

## 1. Introduction

Head and neck cancer (HNC) is a challenging form of cancer affecting areas such as the back of the throat, base of the tongue, and tonsils. It represents a prevalent category within HNC and is characterized by its aggressive nature, often showing a tendency for local spread and widespread dissemination [1]. According to the World Health Organization (WHO), the incidence of HNC has been increasing over the past decades, with an estimated 686,070 new cases worldwide in 2020 [2]. The mortality rates for HNC are also relatively high, with the five-year survival rate depending strongly on a combination of factors such as limited access to healthcare, a late-stage diagnosis, limited resources for treatment, and malnutrition related to the disease.

Malnutrition is a common complication in patients with HNC and is associated with poor outcomes, increased morbidity and mortality rates, and a decreased response to cancer treatments, as well as decreased quality of life [3]. Therefore, nutritional assessment and monitoring of nutritional status is of great importance. However, the diagnosis of malnutrition in patients with HNC can be challenging due to the complex pathophysiology of the disease and the various factors that can contribute to malnutrition, such as dysphagia, mucositis, and treatment-related toxicities [4]. Great effort is now being conducted to establish potential tools to diagnose malnutrition in specific types of diseases, which is of great importance in managing patients with cancer. Traditional techniques for assessing nutritional status, including body mass index (BMI), fat percentage, and serum albumin levels, may not be sensitive enough to detect malnutrition in patients with HNC [5]. Several studies have shown that malnutrition is associated with poor outcomes in patients with head and neck cancer [6,7,8]. A recent systematic review and meta-analysis found an overall positive effect of nutritional intervention during therapy in patients with HNC on body weight [9]. Bao et al. (2020) found that malnutrition in patients with HNC could predict an unfavorable response to chemotherapy [10]. However, there are only a few studies assessing tools to predict and diagnose malnutrition in these patients.

Overall, there is a growing need for the development and implementation of novel techniques to improve the diagnosis of malnutrition in patients with HNC. These techniques should be non-invasive, easily accessible, and able to provide accurate and reliable information about the nutritional status of patients with HNC. Additionally, these techniques should be able to monitor changes in nutritional status over time, as patients with HNC may experience fluctuations in their nutritional status throughout their treatment course [7]. Therefore, improving the diagnosis and management of malnutrition in patients with HNC is crucial to improving survival rates and reducing morbidity and mortality.

In our study, we hypothesize that using new tools to diagnose malnutrition in patients with HNC could predict potential outcomes to improve survival rates and quality of life. Therefore, our aim was to assess the use of ultrasound techniques to predict the nutritional status, malnutrition, as well as other cancer outcomes, such as complications and survival rates. We also aimed to validate the ultrasound techniques against other classical methods to better understand the functional analysis of ultrasound on the nutritional status of patients with HNC.

## 2. Materials and Methods

### 2.1. Study Design

This cross-sectional observational study recruited patients with HNC from the nutrition unit of the Endocrinology and Nutrition Unit of various hospitals in Andalusia, Spain (“Virgen de la Victoria” University Hospital and Malaga Regional Hospital (Málaga, Spain), “San Cecilio” Hospital and “Virgen de las Nieves” University Hospital (Granada, Spain), “Puerta del Mar” University Hospital and Jerez de la Frontera University Hospital (Cádiz, Spain), Jaen University Hospital (Jaen, Spain), “Virgen del Rocio” University Hospital and “Virgen de Valme” University Hospital (Seville, Spain), “Reina Sofía” University Hospital (Córdoba, Spain), Badajoz University Hospital (Badajoz, Spain)) between 2021 and 2022. A total of 494 patients diagnosed with HNC at different stages were included in the study. The diagnosis was confirmed through medical records and pathological examinations, and biopsy samples were classified by pathologists according to the histological features, and the “World Health Organization Classification of Tumors of the Digestive System” (2016) [11]. All patients were included in the first two weeks of radical radiotherapy treatment with chemotherapy and other systemic treatments. All patients had at least a 1-year follow up. This follow-up entailed a clinical visit every three, six, and twelve months for the first year. The inclusion criteria for the study were patients with HNC who had received no more than 2 weeks of radiotherapy treatment and had agreed to participate in the study by providing informed consent. Patients were excluded from the study if they declined to undergo measurements using a bioelectrical impedance analysis (BIA) due to reasons related to ethnicity, extensive skin lesions, the extravasation of fluids through the route and local hematomas, amputation, or a life expectancy of less than 3 months. All subjects provided their informed consent before participating in the study, which was reviewed and approved by the Ethics Committee of the “Virgen de las Nieves” University Hospital (reference code: 2381-M1-22).

### 2.2. Biochemical Variables

We conducted measurements of distinct biomolecular markers related to nutrition and inflammation, including prealbumin and the *C*-reactive protein (CRP)/prealbumin ratio. Prealbumin, unlike albumin, proves to be highly sensitive in detecting alterations in overall body protein levels and remains unaffected by hydration status [12]. Its correlation with CRP levels, a marker solely indicative of inflammation in the body, enhances its potential as an indicator for predicting both morbidity and mortality, reflecting changes in nutritional and inflammatory conditions [13,14]. Moreover, the CRP/prealbumin ratio demonstrates an independent correlation with hospital mortality [15].

### 2.3. Anthropometric and Body Composition Measurements 

A body composition analysis was conducted using a 50 kHz phase-sensitive impedance analyzer (BIA 101 Whole Body Bioimpedance Vector Analyzer, AKERN, Florence, Italy) with tetrapolar electrodes positioned on the right hand and foot, delivering 800 µA. The measurements were taken while the patient was in a supine position on a hospital bed to ensure stability, as positional changes can affect certain values. A five-minute rest period in the supine position was observed before measurements to minimize the impact of fluid shifts caused by changes in posture. The bioelectrical impedance vector analysis (BIVA) focused on the position of the impedance vector on the R/Xc graph relative to tolerance ellipses generated from a reference population, emphasizing R and Xc values normalized by body height [16]. Measurements were standardized based on sex and age using data from healthy Italian adults [17,18]. The phase angle (PA) was calculated as arctan (Xc/R) × (180°/π) [16,17], with an individual standardized PA value (SPA) derived by comparing the patient’s PA value with the reference population, adjusted for age and sex [19]. The accuracy of the BIA instrument was verified daily using a precision circuit provided by the manufacturer, consistently displaying values close to the 385 Ohm reference value. In vivo reproducibility of BIA measurements showed low coefficients of variation (CV) ranging from 1–2% for R and Xc.

Further variables were derived from the bioelectrical impedance analysis (BIA), encompassing bioimpedance-derived parameters related to hydration (fluid percentage within the fat-free mass (FFM) values) and nutritional status (creatine excretion rate measured in mg/kg/24 h, obtained from BCM values). All bioimpedance measurements were conducted while patients were in a supine position on a bed. The data obtained from BIA included categorizations such as FFM (kg) and the FFM index (FFMI) (%); fat mass (FM) (kg) and the FM index (FMI) (%); muscle mass (MM) (kg); skeletal muscle mass (SMM) (kg) and the SM index (SMI) (%); intracellular water (ICW); total body water (TBW) (kg); and body cell mass (BCM) (kg). Precise height measurements were taken using a 2 mm sensitivity laser height rod. Data collection was overseen by a dietitian at both the initiation and conclusion of the study.

Additionally, handgrip strength (HGS) was assessed using a JAMAR hand dynamometer (Asimow Engineering Co., Los Angeles, CA, USA). The measurement involved evaluating grip strength while seated, with the dominant elbow flexed at a 90-degree angle. Patients were instructed to perform three maximum isometric contractions, allowing brief pauses between measurements, and the median value was recorded. Functional assessments, such as the Test Up and Go, were administered while the patient was seated in a chair. This test involved measuring the time taken to rise, walk 3 m, turn around, walk an additional 3 m, and return to a seated position, all timed in seconds. Similarly, a 6 min walk test was conducted to evaluate the patient’s endurance, measuring the distance covered within a span of 6 min [20].

### 2.4. Muscle and Adipose Tissue Muscle Ultrasound Assessment 

Muscle ultrasonography was conducted on the quadriceps rectus femoris (QRF) of the lower extremity using a 10 to 12 MHz probe and a multifrequency linear matrix (Mindray Z60, Madrid, Spain). This examination was performed on all subjects while they were in a supine position. The assessment was carried out without applying compression, positioned at the lower third from the superior pole of the patella and the anterior superior iliac spine. Measurements included assessing anteroposterior muscle thickness, circumference, and cross-sectional area [21]. The ultrasonography procedure was performed by an individual previously trained in this technique. The probe was positioned perpendicular to the longitudinal and transverse axes in the QRF to measure parameters such as the rectus femoris cross-sectional area (RF-CSA), rectus femoris circumference (RF-CIR), RF-axis (-X-axis and -Y-axis), and L-SAT (subcutaneous fat of the leg) [22]. The same trained individual conducted the ultrasonography, performing three measurements for each parameter, and the mean value was calculated.

For assessing adipose tissue, the midpoint between the xiphoid appendix and the navel was identified for imaging in the abdominal area. Here, measurements were taken for T-SAT (total subcutaneous abdominal fat), S-SAT (superficial subcutaneous abdominal fat), and VAT (Preperitoneal or Visceral Fat) in centimeters [23]. The global adipose tissue (GAT) and GAT index (GATi) were calculated by summing T-SAT, L-SAT, and VAT, and T-SAT, L-SAT, and VAT divided by height, respectively.

### 2.5. Assessment of Nutritional Status in Patients with Oropharyngeal Cancer

To diagnose malnutrition based on the GLIM criteria, the following diagnostic criteria for moderate and severe cases were used: moderate cases included a BMI < 20 and age < 70, or FFMI < 17 in males with weight loss between 5% and 10%, and a BMI < 22 and age ≥ 70, or FFMI < 15 in females with weight loss between 5% and 10%. Severe cases were diagnosed with a BMI < 18.5 and age < 70 with weight loss greater than 10%, and a BMI < 20 and age ≥ 70 with weight loss greater than 10%. The European Working Group on Sarcopenia in Older People’s diagnostic criteria for sarcopenia were used to assess decreased muscle strength, with a cut-off of <27 kg in men and <16 kg in women [24]. The Patient-Generated Subjective Global Assessment (PG-SGA) was conducted [25]. A trained nutritionist classified patients by SGA into one of three categories: (A) well nourished; (B) moderately malnourished; or (C) severely malnourished.

### 2.6. Statistical Analysis

The results are presented as the mean ± standard deviation (SD) for continuous variables and as a number (percentages) for categorical variables. A Student *t*-test or Wilcoxon test was performed according to the normality of the variables included in this study. Pearson correlation coefficients between variables were used. Linear and logistic regression analyses were included in this study. The odds ratio (OR) (95% confidence intervals (CIs)) was obtained using a logistic regression analysis. The evaluation of the predictive property of muscle mass variables was based on the receiver operating characteristic (ROC) curve and AUC. A nomogram model for predicting mortality risk was developed and validated based on the results of a multivariable COX regression analysis using the rms package and foreign package in R software. A decision tree was created using the rpart package, and random forest was implemented using Randomforest package analyses, and graphic representation was pointed out, performed using R v. 3.5.1 software (Integrated Development for R. RStudio, PBC, Boston, MA, USA), and the significance *p* value was set using a *p* < 0.05 test, or the Chi-squared test.

## 3. Results

### 3.1. General Characterization of the Population Study

A summary of the general characteristics of the population study, including demographic variables, assessment methods, biochemical variables, and clinicopathological variables, is shown in Table 1. Due to the differences in muscle mass and fat deposits and distribution between males and females, we divided our population by the sex variable and treated females and males as separate groups for personalized nutritional assessment in clinical practice. Regarding demographic variables, we did not observe a significant difference in age or weight loss (%). However, males had higher weight (*p* < 0.001) and BMI (*p* = 0.011) and lower weight loss (*p* = 0.008) compared to females (Table 1 and Supplementary Table S1).

Regarding BIA variables, PA and SPA were higher in males than in females (both with *p* < 0.001). In addition, Xc and Rz were lower in males than in females (both with *p* < 0.001). For adipose tissue variables, FM and FMI did not differ between the two groups. As for muscle mass variables, FFMI, SMI, MM, SMM, ASMM, and FFM were higher in males than in females (all with *p* < 0.001). For water content variables, BCM, BCMI, TBW, ECW, ICW, and BCM were also higher in males than in females (all with *p* < 0.001) (Table 1 and Supplementary Table S1).

When we focused on the study of muscle mass, males had higher hand grip strength than females (*p* < 0.001). Next, we used the ultrasound tool to evaluate muscle mass (quadriceps echography). We found that the RF-CSA, RF-CIR, RF-X-axis, and RF-Y-axis were significantly higher in males than in females (all with *p* < 0.001). Regarding variables of adipose tissue (quadriceps and abdominal echography), we found that L-SAT (*p* < 0.001), T-SAT, and S-SAT (*p* = 0.008 and *p* = 0.001, respectively) were higher in females than in males. As for GAT and GATi, females had higher values when compared to males (*p* = 0.015 and *p* = 0.001). Nevertheless, no significant difference was found in VAT when comparing the two groups (Table 1). As for biochemical variables, we found that creatinine and prealbumin were higher in males than in females (*p* < 0.001 and *p* = 0.002, respectively), while total cholesterol was lower in males than in females (*p* = 0.003) (Table 1). Complete information regarding additional variables is summarized in Table 1 and Supplementary Table S1.

### 3.2. Association between Ultrasound Assessment Methods with Nutritional Status

**Figure 1:**
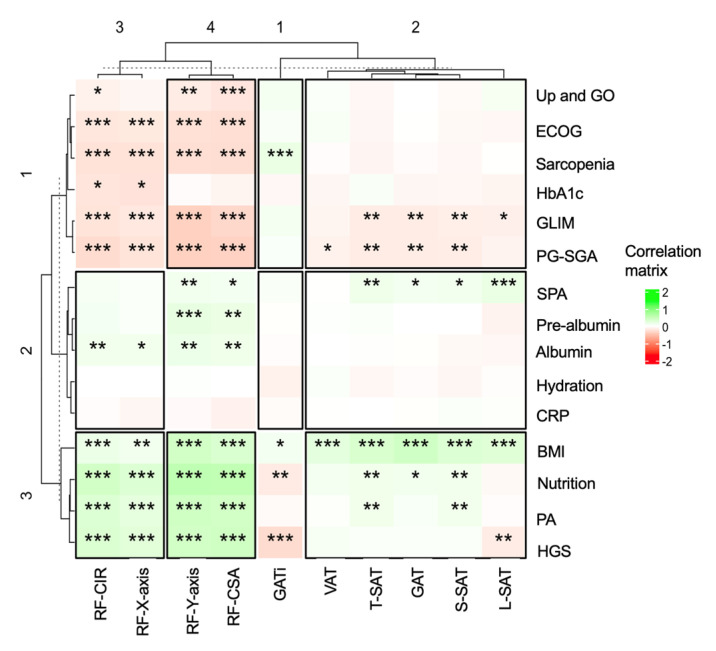
Correlation plot of ultrasound tools (X-axis) and nutrition variables (Y-axis). Horizontal (1, 2, 3) and vertical (1, 2, 3 and 4) refer to clusters created due to data similarity. Pearson’s correlation or Point-biserial coefficients between variables were used and asterisk indicates significant correlation between variables according to the Pearson’s or Point-biserial correlation test (* *p* < 0.05; ** *p* < 0.01; *** *p* < 0.001).

We next investigated the relationship between ultrasound assessment methods and nutritional status variables in patients with HNC. We performed a Point-biserial correlation analysis for categorical variables and a Pearson’s correlation analysis for continuous variables (Figure 1).

When we focused on studying muscle mass, we found that these variables (RF-CSA, RF-CIR, RF-X-axis, and RF-Y-axis) were positively associated with all variables in the first cluster, concerning health status (BMI, nutrition, PA, and HGS) (all with *p* < 0.001). Concerning adipose tissue (composed of GATi, L-SAT, S-SAT, GAT, T-SAT, and VAT), we found that all of them were associated with BMI (most with *p* < 0.001). However, L-SAT and GATi were negatively correlated with HGS (*p* < 0.01 and *p* < 0.001) (Figure 1). Regarding the second cluster, concerning inflammation variables (composed of SPA, prealbumin, albumin, hydration, and CRP), we found that muscle mass variables were positively associated with albumin and partially associated with prealbumin (all with *p* < 0.001), while adipose tissue variables were mostly negatively associated with SPA (most with *p* < 0.001). In the third cluster concerning malnutrition outcomes (Up and Go, ECOG, sarcopenia, HbA1C, GLIM, and PG-SGA), we found that muscle mass variables were negatively associated with all malnutrition variables (all with *p* < 0.001), while adipose tissue variables were positively associated with GLIM and PG-SGA (most with *p* < 0.01) (Figure 1).

Finally, we studied the association between ultrasound tools and HNC outcomes. We found that muscle mass variables were mostly and negatively associated with mortality and hospital admission (most with *p* < 0.01), whereas adipose tissue variables were correlated to cancer complications and palliative variables (most with *p* < 0.05) (Supplementary Figure S1).

### 3.3. Regression Models to Predict Malnutrition Using Ultrasound Methods

Furthermore, we evaluated the ability of ultrasound measurement variables to predict the risk of malnutrition (determined with GLIM criteria and PG-SGA) and the risk of developing sarcopenia using a multivariate logistic regression analysis (Table 2). After adjusting for age, sex, and BMI (with a cut-off of BMI lower than 22 kg/m^2^, for patients over the age of 70, and BMI lower than 20 for patients under the age of 70), we found that high levels of the RF-CSA (OR = 0.81 (0.68–0.98), *p* < 0.05) and RF-Y-axis (OR = 0.31 (0.15–0.61), *p* < 0.001) were associated with a decreased risk of malnutrition, as defined with GLIM criteria. Next, we examined the association between ultrasound measurements and PG-SGA. The variables were adjusted for age, sex, and BMI as a categorical variable. The BMI cut-off was set for patients with a BMI lower than 20 to minimize the effect of patients with malnutrition. We found the same pattern as observed for the GLIM criteria. High levels of the RF-CSA (OR = 0.78 (0.62–0.98), *p* < 0.05), RF-CIR (OR = 0.79 (0.63–0.99), *p* < 0.05), and RF-Y-axis (OR = 0.39 (0.17–0.88), *p* < 0.05) were associated with a decreased risk of malnutrition, as defined with PG-SGA (Table 2). Finally, when we focused on studying the risk of sarcopenia, we adjusted the variables for age, sex, and BMI (with a BMI cut-off set at 25 for patients). We found that high levels of the RF-CSA (OR = 0.64 (0.49–0.82), *p* < 0.001), RF-CIR (OR = 0.74 (0.60–0.91), *p* < 0.01), RF-X-axis (OR = 0.42 (0.24–0.73), *p* < 0.01), and RF-Y-axis (OR = 0.27 (0.11–0.68), *p* < 0.01) were associated with a decreased risk of sarcopenia (Table 2).

As none of the adipose tissue variables were associated with GLIM, PG-SGA, or sarcopenia, we examined the relationship between adipose tissue variables and BMI, FMI, and FM. Our findings indicate that BMI, FMI, and FM were significantly associated with all adipose tissue variables (L-SAT, T-SAT, S-SAT, VAT, GAT, and GATi) (all with *p* < 0.001) (see Supplementary Table S2). These results confirm that the ultrasound method of measuring adipose tissue is highly correlated with other methods of assessing fat deposition and distribution. Additionally, since inflammation can impact the distribution of fat, we examined the connection between inflammatory variables, including CRP, CRP/albumin, prealbumin, and NAK, and adipose tissue variables. Nevertheless, we did not discover any correlation between inflammatory variables and adipose tissue ultrasound variables (refer to Supplementary Table S3 for details).

### 3.4. Regression Models to Predict Oropharyngeal Cancer Outcomes Using Ultrasound Methods

To better understand the relationship between ultrasound assessment methods and outcomes in patients with advanced cancer, we conducted a multivariate logistic regression analysis to determine whether ultrasound assessment methods can predict the risk of mortality, complications, hospital admission, and palliative care (refer to Table 3 for details). For the analysis of mortality risk, we adjusted the assessment variables for age, sex, and BMI. We found that high levels of the RF-CSA (OR = 0.76 (0.59–0.98), *p* < 0.05), RF-X-axis (OR = 0.57 (0.33–0.94), *p* < 0.05), and RF-Y-axis (OR = 0.33 (0.12–0.83), *p* < 0.05) were associated with a decreased risk of mortality. However, we did not observe any correlation between adipose tissue ultrasound variables and mortality risk. Next, we examined the association between ultrasound measurements and complications in patients with advanced cancer. After adjusting for age, sex, and BMI, we found that T-SAT (OR = 0.39 (0.16–0.94), *p* < 0.05) was associated with a decreased risk of developing cancer complications. Regarding the probability of being hospitalized, we observed a similar pattern to the mortality analysis. After adjusting for age, sex, and BMI, we found that high levels of the RF-CSA (OR = 0.78 (0.63–0.96), *p* < 0.05) and RF-X-axis (OR = 0.59 (0.37–0.91), *p* < 0.05) were associated with a decreased risk of hospitalization. Adipose tissue ultrasound variables did not correlate with the risk of hospitalization. Furthermore, we did not find any correlation between nutritional ultrasound variables and palliative care.

**Figure 2:**
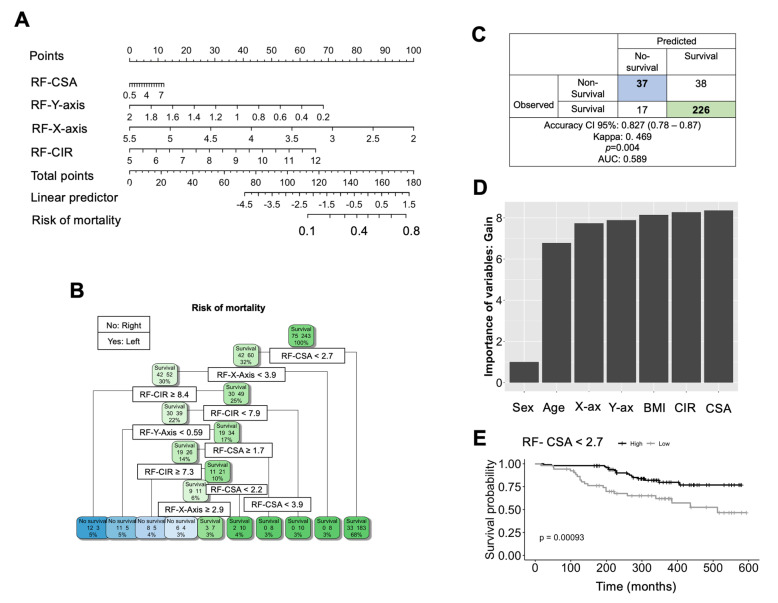
Random forest and machine learning analysis. (**A**) A nomogram of ultrasound measurements predicting overall mortality. (**B**,**C**) Decision tree and table calculate the precision of the model, predicting overall survival. (**D**) Random forest evaluating the most important variable. (**E**) Overall survival using RF-CSA as cut-off of 2.7.

Important decision tree, random forest, and relative ultrasound assessment variables were used to predict mortality in patients with oropharyngeal cancer. According to the results of the multivariate multiple logistic regression analysis, the RF-CSA, RF-CIR, RF-X-axis, and RF-Y-axis were incorporated into the nomogram model to predict the risk of mortality for patients with ORL cancer using ultrasound variables. The nomogram was developed to predict the probability of mortality at 600 days. The obtained C-index for the nomogram was 0.704, and the Brier score was 16.8 (see Figure 2A).

Next, we developed a decision tree to select variables for predicting the risk of mortality (Figure 2B). The decision tree for mortality binary classification contained a sequence of nodes that could classify the risk of mortality based on the ultrasound assessment method, with a focus on muscle mass. In this model, we classified all study samples. We found that the RF-CSA, -CIR, and -X-axis were the top three variables that played an important role in predicting outcomes. In this model, RF-CSA contributed the most power to classification and was selected as the primary node. With RF-CSA < 2.7 as a split, the model classified up to 68% of patients as possessing a high probability of survival and 32% with low survival probability. This cut-off value was confirmed with a predictive study, which was 2.6 with an AUC of 0.653 (0.59–0.77) (see more details in Supplementary Table S4, which contains all muscle mass ultrasound values). For patients with a value of RF-CSA > 2.7, after several nodes containing variables such as -CIR, -Y-axis, and -X-axis, the following was the case. On this side, for patients with a value of RF-CSA < 2.7, after several nodes containing ultrasound variables, an RF-X-axis < 3.9 and RF-CIR ≥ 8.4 predicted up to 3% and 5% of non-survival patients, respectively. Additionally, RF-CIR < 7.9 and an RF-Y-axis < 0.59 predicted up to 3% and 5% non-survival patients, respectively. The model’s accuracy for predicting mortality, including a good prediction for non-survival/survival (37/17) and survival/non-survival (226/38) patients, was 82.7% (95% CI: 0.78–0.87) with a value of Kappa of 0.469 and *p* < 0.001, and a predicted AUC of 0.589 (Figure 2C).

The mean decrease accuracy (MDA) calculated with the random forest showed that information gain (or node purity) is a measure of the usefulness of a split at a particular node (the income graph is represented in Supplementary Figure S1A). Most of the variables contributed to the model’s power for classification, including the importance of variables RF-CSA, RF-CIR, and RF-X- and Y-axis, adjusted for age, sex, and BMI. We found that this model was consistent with the decision tree model, with RF-CSA being the most important variable (Figure 2D). We also studied the overall survival of patients with HNC with high RF-CSA, which was higher than that of patients with a low RF-CSA value, according to the cut-off value of 2.7. We found that patients with a low RF-CSA value lower than 2.7 had worse survival rates than those with a higher value of RF-CSA, higher than 2.7 (*p* < 0.001) (Figure 2E). The survival rate regarding the RF-Y-axis is represented in Supplementary Figure S2B.

## 4. Discussion

The present study aimed to investigate the nutritional status of patients with HNC using ultrasound assessment methods and to explore their association with demographic, clinicopathological, and biochemical variables, as well as the utility to predict malnutrition and overall survival. Our findings demonstrated that males and females with HNC have different body composition profiles, and personalized nutritional assessment and intervention should be considered for each sex. In addition, we found that ultrasound assessment methods were able to predict malnutrition and sarcopenia in patients with HNC, indicating that these techniques could be useful in clinical practice. Furthermore, the ultrasound techniques were able to predict the risk of mortality, supported by the random forest and decision tree algorithms identifying RF-CSA as the most important variable for predicting mortality risk with accuracy. Our study potentially positions muscle mass assessment using ultrasound methods as a valuable tool in clinical practice for predicting malnutrition and improving survival rates in patients with HNC.

The introduction of ultrasound tools in clinical practice not only enhances current medical models, such as classical anthropometry and vector bioelectrical impedance, but also provides support to better estimate the risk of malnutrition. This opens new possibilities for clinical practice, particularly in comparison to more complex and less accessible techniques, such as DEXA.

Our analysis revealed that the distribution of muscle mass and fat is different in males and females, emphasizing the importance of accounting for sex when assessing morphofunctional parameters. Males generally have more muscle mass and less body fat than females with HNC, even after accounting for differences in body size and weight. These findings are consistent with previous studies that have reported sex differences in body composition and metabolism [26,27,28]. Sex-specific reference values can aid in identifying individuals who are at a higher risk for health issues related to muscle mass and fat deposits, such as malnutrition and sarcopenia in patients with cancer. This can help increase overall survival and personalize the management of patients with cancer, underscoring the importance of addressing sex bias in malnutrition management.

Specifically, we found that muscle mass ultrasound measurements, such as the RF-CSA, FR-CIR, RF-X-axis, and RF-Y-axis, were more strongly associated with malnutrition (as defined with GLIM criteria and PG-GSA) and sarcopenia in patients with HNC, as shown in Figure 1, and in the logistic regression analyses adjusted for age, sex, and BMI, shown in Table 2. However, adipose tissue ultrasound measurements were only weakly associated with malnutrition and not with sarcopenia, indicating that muscle mass is a more important indicator of overall health and nutritional status. Several studies have shown that low muscle mass is strongly associated with malnutrition in various health and disease conditions [29,30]. Malnutrition and sarcopenia are common complications in patients with cancer, which can negatively impact survival outcomes. Hence, the early detection of these conditions and understanding nutritional requirements could lead to personalized nutritional interventions to attenuate and prevent the loss of muscle mass in patients with cancer and maximize muscle recovery [31]. Therefore, muscle mass ultrasound measurements could be a useful tool for predicting malnutrition and sarcopenia in patients with HNC. In our study, patients may have experienced both sarcopenia and cachexia. While defining cancer-related cachexia is complex, we refer to sarcopenia because all patients meet its criteria, allowing us to assume its presence, unlike cachexia.

When we focused on the study of ultrasound assessment and HNC outcomes, we still found that muscle mass variables were associated with mortality and hospital admission, as shown with a systematic review conducted by Casey et al. (2022) [32]. In this study, the authors found that muscle mass variables showed significant association with length of stay, readmission, and survival rates. Another study also found that ultrasound measurements of muscle mass were useful to assess low muscularity at ICU admission [33] and muscle atrophy in patients who are critically ill [34]. Therefore, muscle mass ultrasound assessment can provide valuable information for clinicians to personalize treatment plans and optimize cancer management. The implementation of point-of-care ultrasound emerges as a valuable tool for guiding personalized nutritional interventions and exercise therapy by providing individualized data on the distribution of muscle mass and adipose tissue, a level of detail unattainable with classical techniques.

Given that muscle mass variables were more correlated to mortality, we decided to focus on the study of these variables to predict the risk of mortality in patients with HNC. We developed and validated a nomogram model based on the results of the multivariate analysis. The *C*-index and calibration curve were used to evaluate the performance and prediction accuracy of the nomogram model, which had a *C*-index of 0.704, indicating high prediction performance. This model provides a more accurate tool to predict mortality in patients with HNC. While several studies have developed nomogram models to predict low muscle mass and sarcopenia in patients with cancer [35,36,37], only a few have related nomograms, muscle mass, and the risk of mortality. Van Vugt et al. (2018) found that their nomogram model had good discriminative performance for mortality risk in patients with liver disease, indicating that it could be useful for clinical practice [38]. To validate our nomogram and determine which muscle variable is most important in this model, we constructed a decision tree using muscle mass ultrasound variables. We found that RF-CSA was the most important variable in our model, with a cut-off value of 2.7, which was able to classify up to 70% of patients with a higher probability of greater survival rates, as validated with the random forest model. Several studies have used a decision tree to predict sarcopenia and mortality in patients with cancer, indicating that it is a useful tool for classifying the importance of variables [39,40,41]. To confirm this observation, we used a Kaplan–Meier analysis to predict survival rates, using the RF-CSA value of 2.7 as a cut-off. We found that values lower than 2.7 were associated with worse overall survival compared to those patients with RF-CSA greater than 2.7. In assessing body composition, while classic tools are cost-effective but less precise in distinguishing between muscle and adipose tissue, ultrasound techniques offer a low-cost advantage with enhanced precision, which is complementary to the classical tools. However, the gold standard methods, such as DEXA and CT, provide unparalleled precision at a higher cost, and are less feasible for routine clinical use, emphasizing the importance of a balanced approach based on specific clinical requirements and resource constraints.

## 5. Conclusions

In conclusion, our study provides important insights into the body composition profile and nutritional status of patients with HNC using ultrasound assessment methods. Our results demonstrate sex differences in body composition and emphasize the importance of personalized nutritional assessment and intervention in clinical practice. Specifically, muscle mass ultrasound nutritional assessment methods are good predictors in identifying patients with HNC at risk of malnutrition and sarcopenia and providing targeted nutritional interventions. Our study also identifies RF-CSA as the most important variable to predict the risk of mortality, indicating that muscle mass assessment is crucial in the management of patients with HNC. Future studies should investigate the effectiveness of muscle mass ultrasound assessment methods in improving the nutritional status and outcomes of patients with HNC.

## Figures and Tables

**Table 1:** Baseline characteristics of the population of study divided by sex.

	All	Males	Females	*p* Value
	*N* = 494	*N* = 386	*N* = 108	
**Demographic variables**				
Age (years)	63.9 (10.1)	64.2 (9.99)	62.8 (10.4)	0.241
BMI (kg/m^2^)	25.2 (4.52)	25.5 (4.34)	24.1 (5.00)	0.011 *
**BIA**				
PA (°)	5.20 (0.91)	5.28 (0.92)	4.91 (0.81)	<0.001 ***
SPA	−0.54 (1.17)	−0.72 (1.09)	0.10 (1.20)	<0.001 ***
BCM (kg)	25.5 (6.11)	27.1 (5.61)	19.7 (4.04)	<0.001 ***
FFMI (%)	18.4 (2.47)	18.9 (2.35)	16.5 (1.93)	<0.001 ***
FMI (%)	6.67 (2.74)	6.58 (2.72)	7.04 (2.82)	0.146
BCMI (%)	9.10 (1.92)	9.45 (1.90)	7.88 (1.44)	<0.001 ***
SMI (cm^2^/m^2^)	8.69 (1.53)	9.22 (1.16)	6.80 (1.09)	<0.001 ***
**Echography exploration**				
RF-CSA (cm^2^)	3.45 (1.29)	3.69 (1.30)	2.65 (0.88)	<0.001 ***
RF-CIR (cm^2^)	8.69 (1.35)	8.91 (1.31)	7.89 (1.16)	<0.001 ***
RF-X-axis (cm^2^)	3.64 (0.55)	3.73 (0.52)	3.34 (0.54)	<0.001 ***
RF-Y-axis (cm^2^)	1.08 (0.37)	1.14 (0.37)	0.86 (0.25)	<0.001 ***
L-SAT (cm^2^)	0.59 (0.30)	0.53 (0.27)	0.82 (0.33)	<0.001 ***
T-SAT (cm^2^)	1.41 (0.59)	1.37 (0.57)	1.57 (0.63)	0.008 **
S-SAT (cm^2^)	0.61 (0.29)	0.58 (0.28)	0.70 (0.29)	0.001 **
VAT (cm^2^)	0.66 (0.74)	0.68 (0.80)	0.58 (0.43)	0.115
GAT (cm^2^)	2.64 (1.14)	2.58 (1.17)	2.91 (0.98)	0.015 *
GATi (cm)	0.14 (0.23)	0.10 (0.05)	0.30 (0.48)	0.001 ***
**Functional measurement**				
HGS max (kg)	32.9 (10.2)	35.7 (9.26)	22.8 (6.48)	<0.001 ***
HGS mean (kg)	31.2 (10.0)	34.0 (9.12)	21.6 (6.56)	<0.001 ***
TUG (s)	8.10 (2.73)	7.93 (2.62)	8.70 (3.04)	0.034 *
**Biochemical variables**				
Glucose (mg/dL)	99.4 (17.2)	99.8 (17.4)	98.0 (16.4)	0.376
Urea (mg/dL)	38.5 (24.6)	38.7 (25.0)	38.1 (23.2)	0.842
Creatinine (mg/dL)	0.82 (0.18)	0.85 (0.18)	0.72 (0.13)	<0.001 ***
Total cholesterol (mg/dL)	187 (41.9)	184 (41.8)	199 (40.1)	0.003 **
TSH (µUI/mL)	2.25 (6.91)	2.26 (7.71)	2.22 (2.90)	0.958
HbA1c (%)	5.98 (0.71)	5.96 (0.68)	6.07 (0.88)	0.611
Proteins (g/dL)	6.99 (0.59)	7.00 (0.61)	6.97 (0.47)	0.557
Albumin (g/dL)	4.00 (0.87)	4.03 (0.95)	3.89 (0.51)	0.070
Prealbumin (mg/dL)	24.4 (7.55)	25.1 (7.92)	22.2 (5.68)	0.002 **
CRP (mg/L)	18.0 (31.1)	18.2 (31.6)	17.0 (29.6)	0.761
**Clinicopathological variables**				
Cancer stage:				0.094
I	34 (7.28%)	26 (7.16%)	8 (7.69%)	
II	39 (8.35%)	28 (7.71%)	11 (10.6%)	
III	97 (20.8%)	78 (21.5%)	19 (18.3%)	
IVA	64 (13.7%)	45 (12.4%)	19 (18.3%)	
IVB	145 (31.0%)	123 (33.9%)	22 (21.2%)	
IVC	88 (18.8%)	63 (17.4%)	25 (24.0%)	
Complications:				0.285
No	161 (37.1%)	130 (38.6%)	31 (32.0%)	
Yes	273 (62.9%)	207 (61.4%)	66 (68.0%)	
Survival:				0.063
No	306	247	59	
Yes	101	72	29	

Data are expressed as mean ± standard deviations or percentage. Groups were divided by sex variable. Complication variables included dermatitis, dysphagia, mucositis, and asthenia. Asterisk indicates significant difference between groups, according to the Mann–Whitney test (Chi-squared test was used for variables expressed as percentage) (*** *p* < 0.001, ** *p* < 0.01, * *p* < 0.05). Abbreviations—BMI: Body mass index; BIA: Bioelectrical impedance analysis; CRP: C-reactive protein; FM: Fat mass; GAT: Global adipose tissue; GATi: GAT index; HGS: Hand grip strength; PA: Phase angle; RF-CIR: Circumference of quadriceps rectus femoris; RF-CSA: Rectus femoris cross-sectional area; SAT: Subcutaneous adipose fat of leg (L), superficial (S), and total (T) abdominal; SMI: Skeletal muscle index; SPA: Standardized phase angle; TUG: Timed Up and Go.

**Table 2:** Multiple logistic regression of ultrasound measurements and the risk of malnutrition in patients with oropharyngeal cancer.

	GLIM	PG-SGA	Sarcopenia
	OR (CI 95%)	OR (CI 95%)	OR (CI 95%)
Muscle mass			
RF-CSA	0.81 (0.68–0.98) *	0.78 (0.62–0.98) *	0.64 (0.49–0.82) ***
RF-CIR	0.85 (0.71–1.01)	0.79 (0.63–0.99) *	0.74 (0.60–0.91) **
RF-X-axis	0.70 (0.45–1.07)	0.85 (0.49–1.43)	0.42 (0.24–0.73) **
RF-Y-axis	0.31 (0.15–0.61) ***	0.39 (0.17–0.88) *	0.27 (0.11–0.68) **
Adipose tissue			
L-SAT	0.61 (0.26–1.41)	0.53 (0.19–1.50)	1.02 (0.36–2.77)
T-SAT	1.04 (0.70–1.54)	0.78 (0.48–1.27)	0.85 (0.51–1.40)
S-SAT	1.29 (0.58–2.85)	0.67 (0.26–1.79)	0.72 (0.26–1.96)
VAT	0.93 (0.63–1.24)	0.90 (0.64–1.26)	1.04 (0.74–1.49)
GAT	0.99 (0.80–1.23)	0.89 (0.69–1.12)	1.02 (0.79–1.31)
GATi	3.86 (0.88–7.07)	1.01 (0.32–8.92)	7.20 (1.32–22.83)

Multiple logistic regression of GLIM, PG-SGA, and sarcopenia and its relationship with ultrasound measurements. All variables were adjusted for age, sex, and BMI (* *p* < 0.05; ** *p* < 0.01; *** *p* < 0.001). To test the effect of malnutrition in the association with ultrasound, in the case of the GLIM, the cut-off of BMI was BMI < 22 in those patients greater than 70 years old, and a BMI < 20 for those patients aged below 70 years old. For the PG-SGA, BMI cut-off was set in those patients that had a BMI lower than 20. In the case of sarcopenia, the cut-off of the BMI was set in those patients that had a BMI lower than 25. Abbreviations—GAT: Global adipose tissue; GATi: GAT index; GLIM: Global Leadership Initiative on Malnutrition; HR: Hazard ratio; RF: Rectus femoris; RF-CIR: Circumference of quadriceps rectus femoris; RF-CSA: Rectus femoris cross-sectional area; PG-SGA: Patient-Generated Subjective Global Assessment; SAT: Subcutaneous adipose fat of leg (L), superficial (S), and total (T) abdominal.

**Table 3:** Multivariate logistic regression of ultrasound measurements and principal oropharyngeal cancer outcomes.

	Mortality	Complications	Hospital Admission	Palliative
	OR (CI 95%)	OR (CI 95%)	OR (CI 95%)	OR (CI 95%)
Muscle mass				
RF-CSA	0.76 (0.59–0.98) *	0.89 (0.72–1.09)	0.78 (0.63–0.96) *	0.93 (0.72–1.21)
RF-CIR	0.88 (0.72–1.08)	0.86 (0.71–1.03)	0.89 (0.74–1.05)	1.09 (0.88–1.36)
RF-X-axis	0.57 (0.33–0.94) *	0.74 (0.47–1.17)	0.59 (0.37–0.91) *	0.84 (0.50–1.42)
RF-Y-axis	0.33 (0.12–0.83) *	0.70 (0.33–1.50)	0.48 (0.22–1.02)	0.77 (0.29–2.00)
Adipose tissue				
L-SAT	0.68 (0.22–2.00)	0.69 (0.29–1.64)	1.97 (0.78–5.11)	0.58 (0.18–1.76)
T-SAT	0.75 (0.43–1.27)	0.66 (0.43–1.02)	0.81 (0.52–1.25)	0.59 (0.32–1.04)
S-SAT	0.93 (0.31–2.74)	0.39 (0.16–0.94) *	0.60 (0.24–1.51)	0.64 (0.19–2.02)
VAT	1.33 (0.59–2.89)	1.53 (0.95–2.91)	0.84 (0.43–1.64)	1.34 (0.55–3.08)
GAT	0.91 (0.60–1.37)	0.96 (0.76–1.22)	1.00 (0.71–1.40)	0.81 (0.52–1.25)
GATi	1.58 (0.53–5.34)	1.92 (0.59–17.72)	1.06 (0.36–3.43)	1.48 (0.36–4.84)

Multivariate logistic regression of overall survival, complications, hospital admission, and palliative care and its relationship with ultrasound measurements. Adjusted by age, gender, and BMI (* *p* < 0.05). Abbreviations—GAT: Global adipose tissue; GATi: GAT index; OR: Odds ratio; RF-CIR: Circumference of quadriceps rectus femoris; RF-CSA: Rectus femoris cross-sectional area; SAT: Subcutaneous adipose fat of leg (L), superficial (S), and total (T) abdominal.

## Data Availability

Data are contained within the article and Supplementary Materials.

## Supplementary Materials

The following supporting information can be downloaded at: https://www.mdpi.com/article/10.3390/nu16030387/s1, Supplementary Table S1. Baseline characteristics of the population of study under the sex variable. Supplementary Table S2. Multiple linear regression of ultrasound measurements and the adipose tissue deposits and distribution. Supplementary Table S3. Logistic regression of ultrasound measurements and inflammatory status in patients with oropharyngeal cancer. Supplementary Table S4. Predictive Value of ultrasound on survival in the population. Supplementary Figure S1. Correlation plot of ultrasound tools (X-axis) and cancer outcomes (Y-axis). Pearson’s correlation or Point-biserial coefficients between variables were used and asterisk indicates significant correlation between variables according to the Pearson’s or Point-biserial correlation test (* *p* < 0.05; ** *p* < 0.01; *** *p* < 0.001). Supplementary Figure S2. (A) Random forest of income evaluating the most important variable. (B) Overall survival using RF-Y-axis according to the median value.
