# Supplementary material for: Ultrasound Muscle Evaluation for Predicting the Prognosis of Patients with Head and Neck Cancer: A Large-Scale and Multicenter Prospective Study
Source: Nutrients. 2024 Jan 29;16(3):387. doi: 10.3390/nu16030387 (PMC10857428; doi:10.3390/nu16030387)
Supplement: Supplementary file 1 [file nutrients-16-00387-s001.zip › nutrients-2830932-supplementary.pdf]

Supplementary Table S1. Baseline characteristics of the population of study under the sex variable

	**All**	**Males**	**Females**	***p* value**
	N=498	***N=390***	***N=108***	
**Anthropometric variables**				
Weight (kg)	70.9 (15.4)	73.7 (14.5)	61.2 (14.7)	<0.001***
Weight loss (%)	6.21 (9.11)	5.40 (7.38)	9.03 (13.2)	0.008**
**BIA**				
Rz (Ω/m)	50.2 (10.7)	49.0 (10.5)	54.3 (10.3)	<0.001***
Xc (Ω/m)	555 (97.4)	533 (85.5)	634 (96.9)	<0.001***
**Fat variables**				
FM (kg)	18.8 (8.07)	18.9 (8.06)	18.5 (8.15)	0.635
**Muscle mass variables**				
MM (kg)	26.1 (6.68)	28.1 (5.62)	18.9 (5.06)	<0.001***
SMM (kg)	26.1 (6.72)	28.1 (5.65)	18.9 (5.09)	<0.001***
ASMM (kg)	19.4 (4.13)	20.7 (3.37)	14.8 (3.09)	<0.001***
FFM (kg)	51.7 (9.25)	54.6 (7.90)	41.4 (5.66)	<0.001***
**Water content variables**				
TBW (kg)	38.1 (7.20)	40.3 (6.20)	30.3 (4.55)	<0.001***
ECW (kg)	19.0 (3.67)	20.0 (3.39)	15.6 (2.24)	<0.001***
ICW (kg)	49.7 (5.47)	50.1 (5.61)	48.4 (4.72)	0.002**
NAK	1.14 (0.24)	1.15 (0.25)	1.11 (0.21)	0.094
**Metabolism and nutrition variables**				
Basal metabolism (kcal)	1476 (223)	1519 (227)	1322 (117)	<0.001***
Hydration (%)	73.8 (2.38)	73.9 (2.43)	73.2 (2.09)	0.301
Nutrition	775 (183)	825 (167)	597 (112)	<0.001**
**Clinicopathological variables**				
				0.677
Chemotherapy				
No		175	50	
Yes		212	58	
Hospital compliance				0.894
No		147	40	
Yes		160	45	
Palliative				0.516
No		234	60	
Yes		72	23	
Progression / Persistence				0.665
No		183	49	
Yes		124	37	

	All N=498	Males N=390	Females N=108	*p* value
Free of disease				0.459
No		222	65	
Yes		84	20	
ECOG				0.370
0		170	45	
1		144	46	
2		22	8	
3		3	2	

Data are expressed as mean ± standard deviations or percentage. Groups were divided by sex variable. Complication variables included dermatitis, dysphagia, mucositis, and asthenia. Asterisk indicates significant difference between groups, according to Mann–Whitney test (Chi-squared test was used for variables expressed as percentage) (***p<0.001, **p<0.01, *p<0.05).

Abbreviations—ASMM: Appendicular skeletal muscle mass; BCM: Body cell mass; BCMI: Body cell mass index; BM: Basal metabolism; ECOG: Eastern Cooperative Oncologic Group; ECW: Extracellular cell water; FFM: Fat-free mass; FFMI: Fat-free mass index; FMI: Fat mass index; MM: Muscle mass; NAK: Sodium and potassium ratio; Rz: Resistance; SSM: Skeletal muscle mass; TBW: Total body water; Xc: Reactance.

Supplementary Table S2. Multiple linear regression of ultrasound measurements and the adipose tissue deposits and distribution

	BMI β (SD)	FMI β (SD)	FM β (SD)
Adipose tissue			
L-SAT	6.53 (0.72)***	4.64 (0.42)***	13.18 (1.26)***
T-SAT	2.99 (0.35)***	2.29 (0.21)***	6.53 (0.61)***
S-SAT	5.84 (0.72)***	4.35 (0.44)***	12.22 (1.29)***
VAT	1.41 (0.29)***	0.93 (0.18)***	2.90 (0.52)***
GAT	1.72 (0.17)***	1.22 (0.10)***	3.62 (0.30)***
GATi	3.55 (1.02)***	2.25 (0.61)***	3.29 (1.84)

Multiple linear regression of BMI, FMI, and FM and its relationship with nutritional ultrasound measurements. Adjusted for age and sex (*p<0.05; **p<0.01; ***p<0.001). **Abbreviations**—BMI: body mass index; FM: fat mass; FMI: fat mass index; SAT: subcutaneous adipose fat of leg (L), superficial (S), and total (T) abdominal.

Supplementary Table S3. Logistic regression of ultrasound measurements and inflammatory status in patients with oropharyngeal cancer

	CRP	CRP/Albumin ratio	Prealbumin	NA/K ratio
	OR (CI 95%)	OR (CI 95%)	OR (CI 95%)	OR (CI 95%)
Adipose tissue				
L-SAT	1.59 (0.56 – 4.56)	0.60 (0.14 – 2.60)	0.48 (0.12 – 1.87)	1.32 (0.41 – 4.47)
T-SAT	0.84 (0.49 – 1.41)	1.01 (0.48 – 2.18)	1.75 (0.90 – 3.55)	0.65 (0.38 – 1.11)
S-SAT	1.37 (0.47 – 3.97)	1.85 (0.38 – 9.91)	1.36 (0.35 – 5.62)	0.85 (0.28 – 2.63)
VAT	1.20 (0.87 – 2.09)	1.03 (0.69 – 2.85)	1.36 (0.82 – 3.75)	0.90 (0.64 – 1.31)
GAT	1.08 (0.84 – 1.44)	1.05 (0.75 – 1.77)	1.18 (0.84 – 1.94)	0.89 (0.68 – 1.16)
GATi	0.75 (0.09 – 2.32)	340 (0.84 – 2860)	26.0 (0.70 – 48826)	0.75 (0.22 – 2.76)

Multiple logistic regression of CRP (cut-off value of 10 mg/dL), CRP/albumin ratio (cut-off value of 1.4), NAK (cut-off value of 1), and ECW/TBW ratio (cut-off value of 0.39). Adjusted by age, gender, and BMI (* $p<0.05$; ** $p<0.01$; *** $p<0.001$). The cut-off of the BMI was set in those patients that had a BMI lower than 25.

Abbreviations—CRP: C-reactive protein; GAT: Global adipose tissue; GATi: GAT index; OR: Odds ratio; NAK: Sodium/potassium ratio; RF-CIR: Circumference of quadriceps rectus femoris; RF-CSA: Rectus femoris cross-sectional area; SAT: Subcutaneous adipose fat of leg (L), superficial (S), and total (T) abdominal

Supplementary Table S4. Predictive Value of ultrasound on survival in the population

	AUC	Cut-off ▴	Sensitivity	Specificity	*p* value
Muscle mass					
RF-CSA	0.653	2.59	77%	55%	0.004
RF-CIR	0.636	7.91	75%	50%	0.014
RF-X-Axis	0.640	3.21	83%	41%	0.013
RF-Y-Axis	0.668	1.09	55%	67%	0.002

Receiver operating characteristic (ROC) for the nutritional ultrasound and survival in patients with OPC. Cut-off involved not adjusting for variables. **Abbreviations**—AUC: area under curve; RF-CIR: circumference of quadriceps rectus femoris; RF-CSA: rectus femoris cross-sectional area.

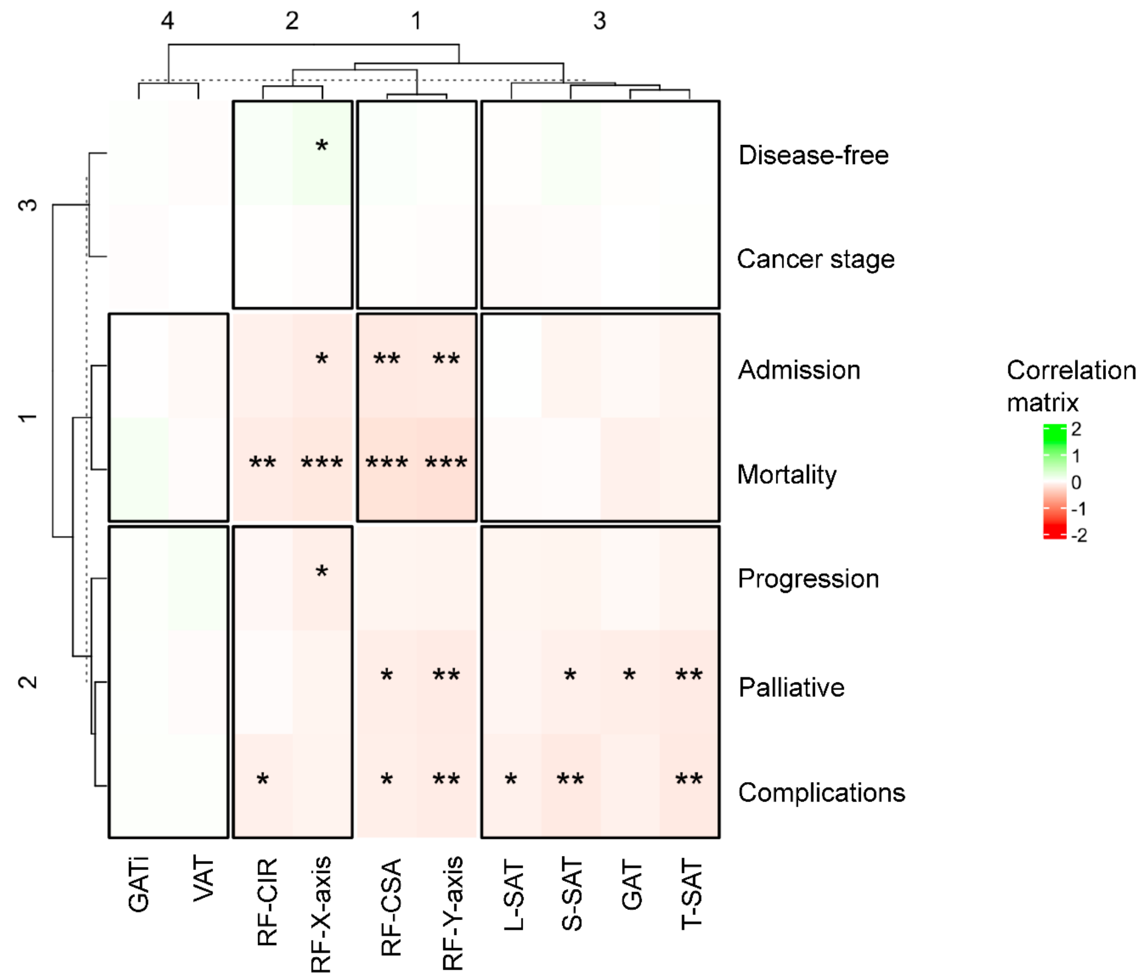

Supplementary Figure S1. Correlation plot of ultrasound tools (X-axis) and cancer outcomes (Y-axis). Pearson's correlation or Point-biserial coefficients between variables were used and asterisk indicates significant correlation between variables according to the Pearson's or Point-biserial correlation test (* $p < 0.05$; ** $p < 0.01$; *** $p < 0.001$).

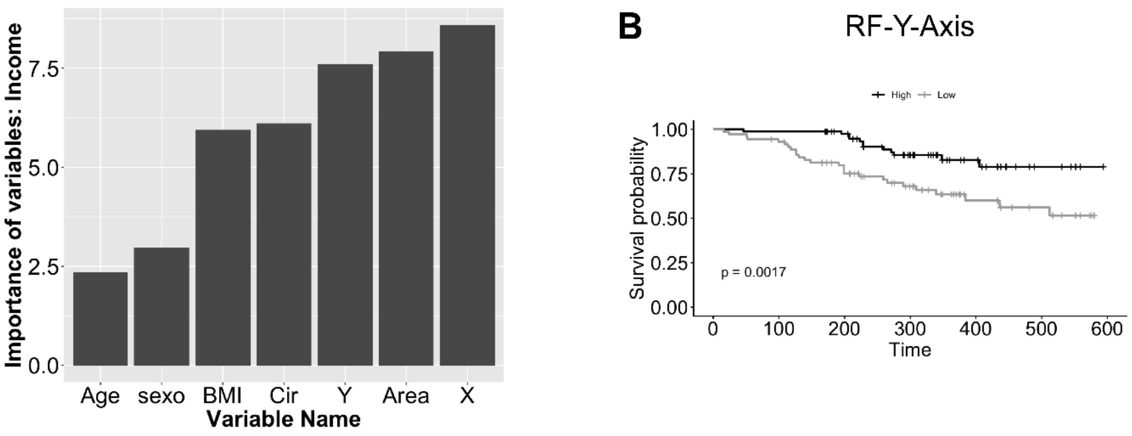

Supplementary Figure S2. (A) Random forest of income evaluating the most important variable. (B) Overall survival using RF-Y-axis according to the median value.
